# Syntheses and crystal structures of benzyl *N*′-[(*E*)-2-hydroxybenzylidene]hydrazinecarboxylate and benzyl *N*′-[(*E*)-5-bromo-2-hydroxybenzylidene]hydrazinecarboxylate

**DOI:** 10.1107/S2056989022009100

**Published:** 2022-09-22

**Authors:** Vinaya, Yeriyur B. Basavaraju, Beliyaiah Lakshmana, Hemmige S. Yathirajan, Sean Parkin

**Affiliations:** a Department of Studies in Chemistry, University of Mysore, Manasagangotri, Mysuru-570 006, India; b Department of Chemistry, University of Kentucky, Lexington, KY, 40506-0055, USA; Moscow State University, Russia

**Keywords:** crystal structure, benzyl­idene hydrazine, benzaldehyde­hydrazone, absolute structure

## Abstract

Benzyl *N*′-[(*E*)-2-hydroxybenzylidene]hydrazinecarboxylate, C_15_H_14_N_2_O_3_ (**I**) and benzyl *N*′-[(*E*)-5-bromo-2-hydroxybenzylidene]hydrazinecarboxylate (**II**), C_15_H_13_BrN_2_O_3_, have been synthesized by the reaction of either 2-hy­droxy­benzaldehyde or 5-bromo-2-hy­droxy­benzaldehyde with benzyl carbazate, respectively. Both the compounds crystallize in the monoclinic crystal system with space groups *Pn* (*Z*′ = 1, **I**) and *P*2_1_/*c* (*Z*′ = 2, **II**). Mol­ecular conformations in each structure are similar, and both structures feature strong intra­molecular O—H⋯N hydrogen bonds, which form *S*(6) ring motifs. There are also strong N—H⋯O and weak C—H⋯O hydrogen bonds in both structures, but their modes of packing within their respective crystals are markedly different. Some comparisons are made with the structures of a few related compounds.

## Abstract 2

The syntheses and low-temperature (90 K) crystal structures of benzyl *N*′-[(*E*)-2-hydroxybenzylidene]hydrazinecarboxylate and benzyl *N*′-[(*E*)-5-bromo-2-hydroxybenzylidene]hydrazinecarboxylate are presented.

## 1. Chemical context

Hy­droxy­benzyl­idene hydrazines exhibit a wide spectrum of biological activities (Sersen *et al.*, 2017 ▸). Benzaldehyde­hydrazone derivatives have received considerable attention for several decades as a result of their pharmacological activity (Parashar *et al.*, 1988 ▸) and photochromic properties (Hadjoudis *et al.*, 1987 ▸). Benzaldehyde­hydrazone derivatives are also important inter­mediates in the synthesis of 1,3,4-oxa­diazo­les, which are versatile compounds with many useful properties (Borg *et al.*, 1999 ▸). Synthesis and biological activities of new hydrazide derivatives (Özdemir *et al.*, 2009 ▸) and biological activities of hydrazone derivatives (Rollas & Küçükgüzel, 2007 ▸) have been reported. In view of the importance of benzyl­idene hydrazines and benzaldehyde­hydrazone derivatives in general, this paper reports the crystal structures of the title compounds, C_15_H_14_N_2_O_3_ (**I**), and C_15_H_13_BrN_2_O_3_ (**II**). 
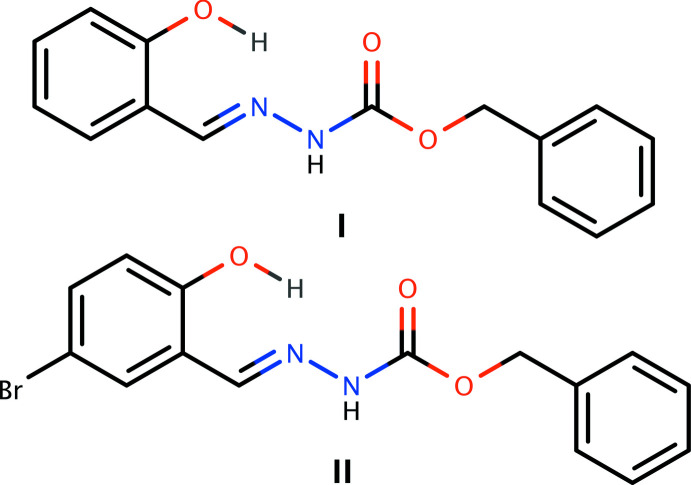


## 2. Structural commentary

**Figure 1:**
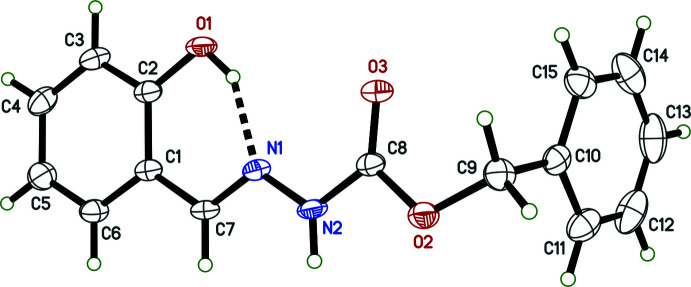
An ellipsoid plot (50% probability) of **I**, showing the intra­molecular hydrogen bond (O1—H1*O*⋯N1) as a dashed line.

**Figure 2:**
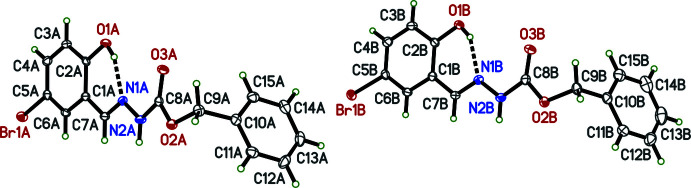
An ellipsoid plot of the asymmetric unit of **II**, showing the intra­molecular hydrogen bonds (O1*A*—H1*AO*⋯N1*A* and O1*B*—H1*BO*⋯N1*B*) as dashed lines.

**Figure 3:**
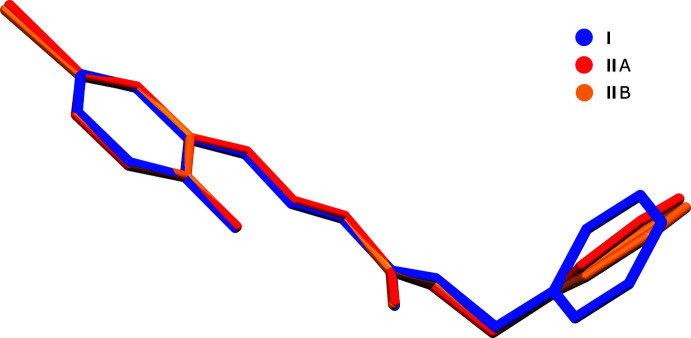
A least-squares fit overlay of **I**, **II** ***A***, and **II** ***B*** showing the similarity of their conformations. That of **I** (blue) differs primarily in the orientation of the benzyl group (right). Diagram generated using *Mercury* (Macrae *et al.*, 2020 ▸).

The mol­ecular structures of benzyl *N*′-[(*E*)-2-hydroxyben­zylidene]hydrazinecarboxylate (**I**) (Fig. 1 ▸) and benzyl *N*′-[(*E*)-5-bromo-2-hydroxybenzylidene]hydrazinecarboxylate (**II**) (Fig. 2 ▸) each consist of a central *N*′-methyl­idene­meth­oxy­carboxyl core flanked by a benzyl group attached to the singly bonded oxygen and a 2-hy­droxy­phenyl (**I**) or 5-bromo-2-hy­droxy­phenyl (**II**) attached to the methyl­idene. There are no unusual bond lengths or angles in either structure. The mol­ecules have strong intra­molecular O—H⋯N hydrogen bonds (Tables 1 ▸ and 2 ▸), forming *S*(6) ring motifs (Etter *et al.*, 1990 ▸). The asymmetric unit of **I** contains a single mol­ecule while that of **II** contains two (labelled *A* and *B* in Fig. 2 ▸). In each case, the [(hy­droxy­phen­yl)methyl­idene]carbohydrazide moieties are essentially planar [r.m.s. deviations 0.0429 Å (**I**), 0.0905 Å (**II** ***A***), 0.0692 (**II** ***B***)]. These form dihedral angles of 79.92 (3)°, 79.74 (4)°, and 74.27 (4)° to the benzyl groups of **I**, **II** ***A***, and **II** ***B***, respectively. Indeed, the V-shaped conformations of **II** ***A***, and **II** ***B*** are strikingly similar, with **I** only deviating to any appreciable degree at the benzyl group, as evidenced by an overlay of the three mol­ecules (Fig. 3 ▸). The conformation of **I** differs from **II** ***A*** and **II** ***B*** primarily by the torsion angles about bonds O2—C9 and C9—C10 (Table 3 ▸).

## 3. Supra­molecular features

In addition to the strong O—H⋯N *intra*mol­ecular hydrogen bonds in **I** and **II**, the structures both feature strong N—H⋯O and weaker C—H⋯O *inter*mol­ecular hydrogen bonds. These inter­actions are summarized in Tables 1 ▸ and 2 ▸. The packing modes are, however, quite different.

**Figure 4:**
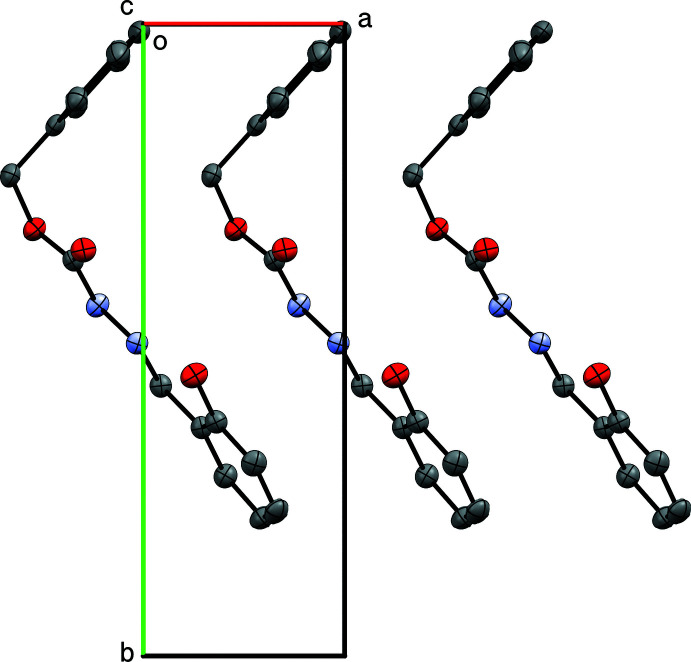
V-shaped mol­ecules of **I** stack into columns parallel to the *a*-axis direction.

**Figure 5:**
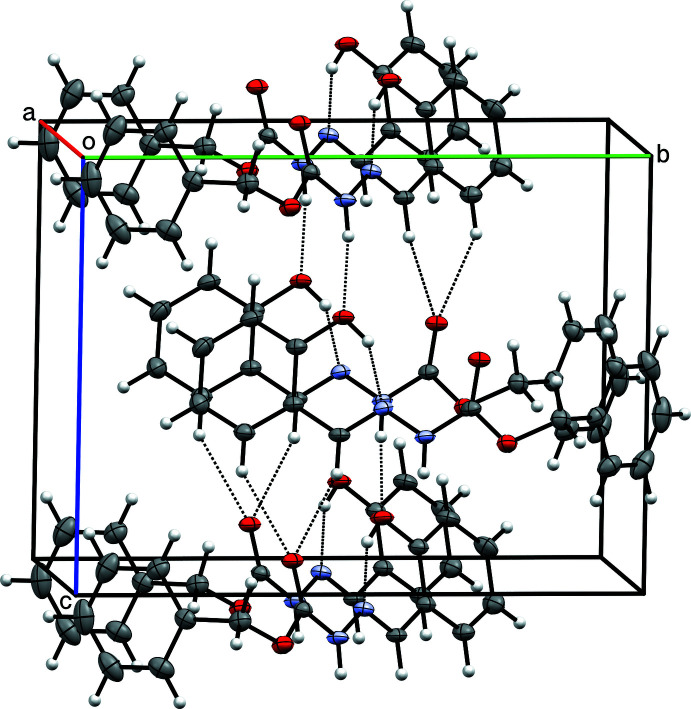
A partial packing plot of **I** showing hydrogen bonding as dashed lines. N—H⋯O and a pair of C—H⋯O (bifurcated) hydrogen bonds link *n*-glide-related mol­ecules into layers parallel to *ac*.

In **I**, the V-shaped (Fig. 3 ▸) mol­ecules stack into columns along [100] (Fig. 4 ▸). These columns inter­act with *n*-glide-related columns *via* the strong N2—H2*N*⋯O1^i^ (symmetry codes as per Table 1 ▸) hydrogen bonds to give *C*(7) chains (Etter *et al.*, 1990 ▸) and with different *n*-glide-related columns *via* the bifurcated C6—H6⋯O3^ii^ and C7—H7⋯O3^ii^ (Table 1 ▸) hydrogen bonds. In combination, these inter­actions produce layers that extend in the *ac* plane (Fig. 5 ▸), which in turn stack along [010].

**Figure 6:**
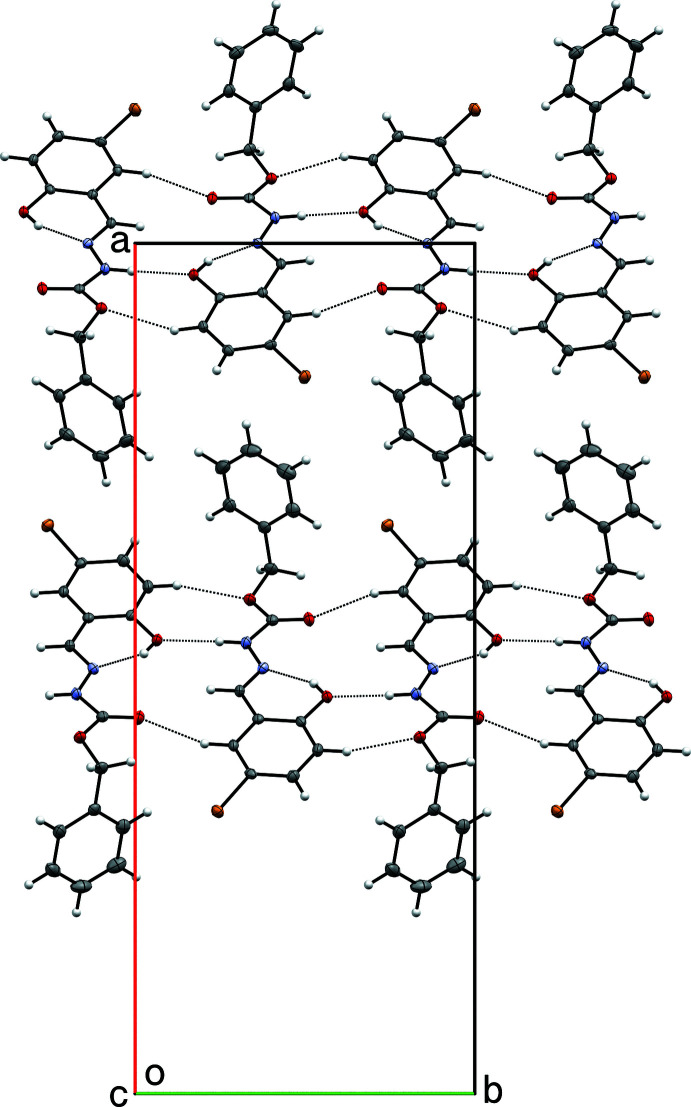
A partial packing plot of **II** showing N—H⋯O and C—H⋯O hydrogen-bonded ribbons along [010] of **II** ***A*** (upper) and **II** ***B*** (lower) mol­ecules.

**Figure 7:**
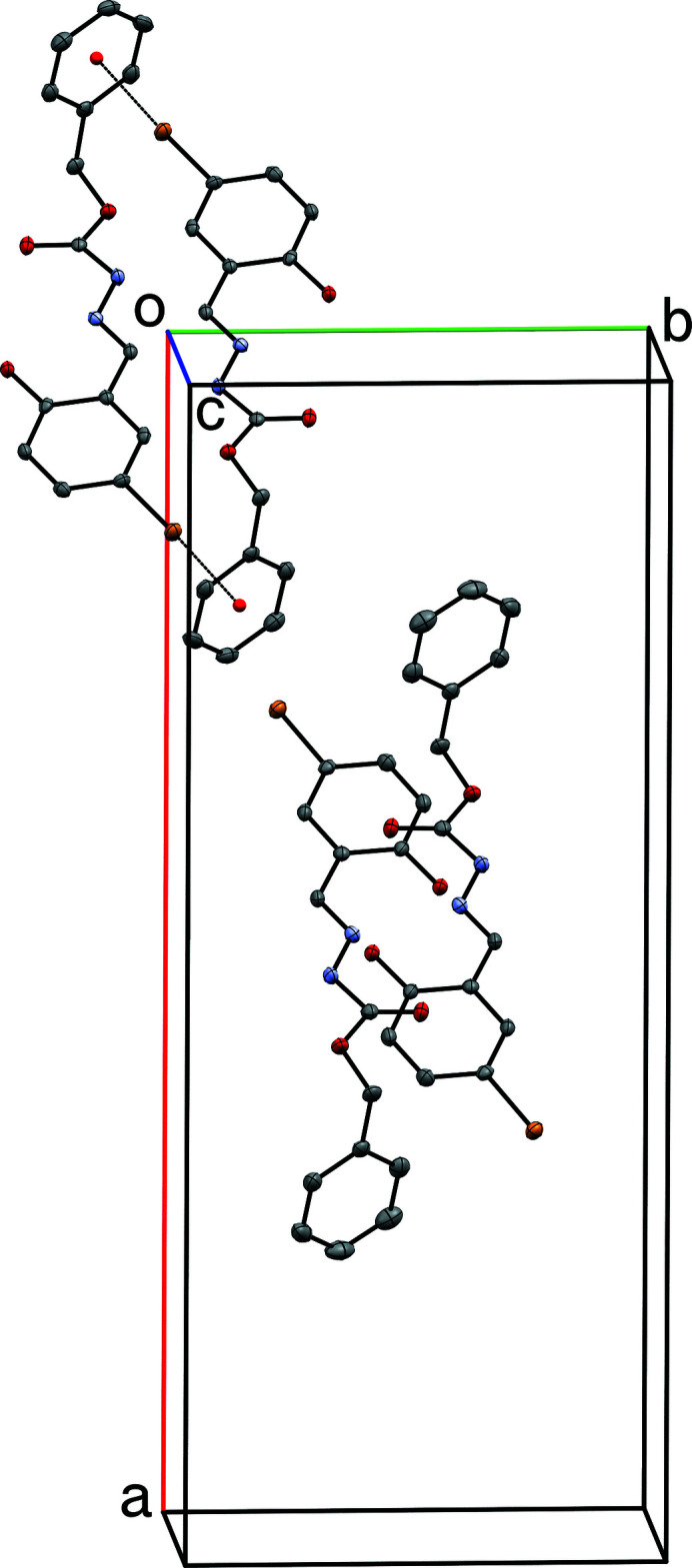
In spite of their similar conformations, inversion-related pairs of **II** ***A*** mol­ecules (upper) are different from inversion-related pairs of **II** ***B*** mol­ecules (lower). For **II** ***A*** there are close contacts between bromine and the inversion-related benzene ring, as shown by the dotted line. No such inter­action exists for **II** ***B***.

In **II**, the independent mol­ecules (*A* and *B*) make hydrogen bonds to 2_1_-screw-related copies of themselves *via* strong (N2—H2*N*⋯O1) and weak (C3—H3⋯O2 and C6—H6⋯O3) hydrogen bonds (Table 2 ▸), forming 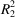 (8) and 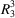 (13) ring motifs (Etter *et al.*, 1990 ▸), leading to adjacent pairs of ribbons that extend along [010] (Fig. 6 ▸). The 5-bromo-2-hy­droxy­phenyl and benzyl groups of **II** ***A*** and **II** ***B*** have notably different environments. For example, inversion-related (−*x*, −*y*, −*z*) pairs of **II** ***A*** mol­ecules have close contacts of 3.3379 (9) Å between their Br1*A* atoms and the centroid of the inversion-related C10*A*–C15*A* ring. There is no corresponding close contact for the **II** ***B*** mol­ecule (Fig. 7 ▸).

**Figure 8:**
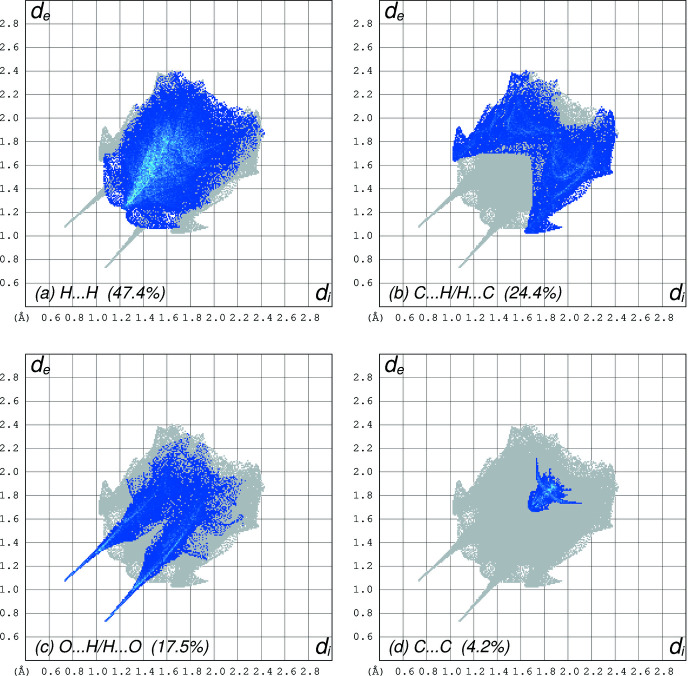
Fingerprint plots obtained from a Hirshfeld surface analysis for **I** using *CrystalExplorer*, separated into (*a*) H⋯H (47.4% coverage), (*b*) C⋯H/H⋯C (24.4%), (*c*) O⋯H/H⋯O (17.5%), (*d*) C⋯C (4.2%). All other contacts are negligible.

The differences in packing are also apparent in the atom–atom contact coverages, as qu­anti­fied by *CrystalExplorer* (Spackman *et al.*, 2021 ▸) fingerprint diagrams (Figs. 8 ▸ and 9 ▸).

## 4. Database survey

A search of the Cambridge Structure Database (CSD, v5.43 with updates as of June 2022; Groom *et al.*, 2016 ▸) for a search fragment consisting of the structure of **I**, but with the two aromatic rings replaced by ‘any group’ gave 340 hits. A fragment including the benzyl group attached to the equivalent of O2 in **I**/**II** gave 105 hits, while a fragment including a phenyl ring at C7 gave 37 hits. A fragment consisting of **I** but without the phenolic OH group gave just four hits: HIXQIQ (Dong & Wang, 2014 ▸), QAVFAY (Shen *et al.*, 2022 ▸), GEZTUD (Chang *et al.*, 2018 ▸) and PIVKUD (Zhang *et al.*, 2019 ▸). In HIXQIQ, a 5-chloro-2-hy­droxy-2-(meth­oxy­carbon­yl)-2,3-di­hydro-1*H*-in­den-1-yl­idene) group is attached to the hydrazine. QAVFAY features a four-membered 1,2-diazete ring, with the phenyl group fluorinated at its 4-position. Structures GEZTUD and PIVKUD each feature pyrazole rings; the former having a 2,2,2-tri­fluoro­ethyl group attached to the pyrazole and a methyl at the 4-position of the phenyl ring, and the latter having a 3,4,5-tri­meth­oxy­phenyl attached to its pyrazole ring.

New Schiff bases derived from benzyl carbazate with alkyl and heteroaryl ketones and crystal structures of benzyl 2-cyclo­pentyl­idenehydrazine­carboxyl­ate (JENFAM, (*E*)-benzyl 2-[1-(pyridin-3-yl)ethyl­idene]hydrazine-1-carboxyl­ate (JENFEQ), (*E*)-benzyl2-[1-(pyridin-4-yl)ethyl­idene]hydrazine­carboxyl­ate (JENFIU) (Nithya *et al.*, 2017 ▸) have also been reported. A selection of other structures similar to **I** and **II** deposited in the CSD are listed in Table 4 ▸.

## 5. Synthesis and crystallization

**Figure 10:**
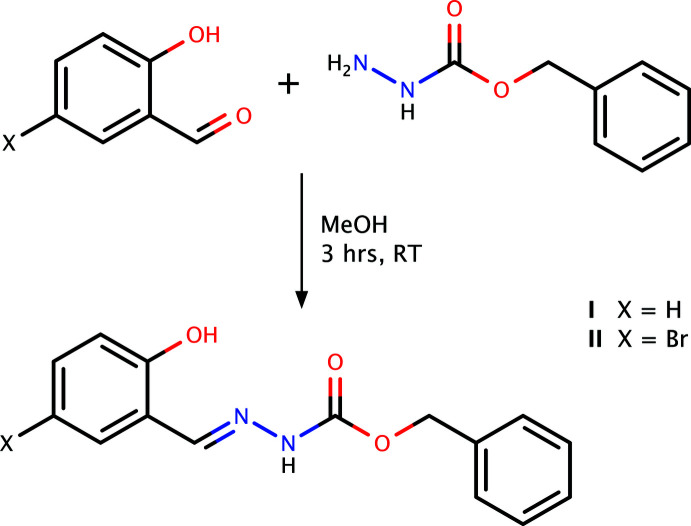
Reaction scheme for the synthesis of **I** and **II**.

Preparation of **I** and **II** followed similar synthetic routes. Either 2-hy­droxy­benzaldehyde (1.2 g, 0.01 mol) (for **I**) or 5-bromo-2-hy­droxy­benzaldehyde (2.0 g, 0.01 mol) (for **II**) and benzyl carbazate (1.66 g, 0.01 mol) were dissolved in methanol (25 ml) and stirred for 3 h at room temperature. The resulting solids were filtered off and recrystallized from ethanol to give **I** and **II** with yields of 80% in both cases. The general reaction scheme is summarized in Fig. 10 ▸. Single crystals suitable for X-ray analysis for both **I** and **II** were obtained by slow evaporation of methano­lic solutions at room temperature (m.p.: 400–402 K for **I** and 468–470 K for **II**).

## 6. Crystal handling, data collection, and refinement

Crystals of **I** and **II** were each secured on the tips of fine glass fibres held in copper mounting pins. The crystal of **I** was mounted from a shallow liquid-nitro­gen dewar using tongs first developed for protein cryocrystallography (Parkin & Hope, 1998 ▸), while the crystal of **II** was mounted directly into a cold-nitro­gen stream. Data for both samples (Cu *K*α for **I** and Mo *K*α for **II**) were collected with the crystals held at 90.0 (2) K. Determination of the absolute structure for **I** was inconclusive *via* traditional full-matrix refinement of Flack’s parameter [*x* = −0.08 (18); Flack & Bernardinelli, 1999 ▸], but Hooft’s Bayesian approach [*y* = 0.00 (8); Hooft *et al.* (2008 ▸), as calculated using *PLATON* (Spek, 2020 ▸)] and Parsons’ quotient method [*z* = 0.04 (10); Parsons *et al.*, 2013 ▸] give credence to the assignment. Refinement progress was checked using *PLATON* (Spek, 2020 ▸) and by an *R*-tensor (Parkin, 2000 ▸). Crystal data, data collection, and refinement statistics are summarized in Table 5 ▸. Carbon-bound hydrogen atoms were included using riding models, with C—H distances constrained to 0.95 Å for C*sp* ^2^H and 0.99 Å for *R*_2_CH_2_. N—H and O—H hydrogen-atom coord­inates were refined. *U*_iso_(H) parameters were set to values of either 1.2*U*_eq_ (C—H, N—H) or 1.5*U*_eq_ (O—H) of the attached atom.

## Supplementary Material

Crystal structure: contains datablock(s) I, II, global. DOI: 10.1107/S2056989022009100/yk2176sup1.cif

Structure factors: contains datablock(s) I. DOI: 10.1107/S2056989022009100/yk2176Isup4.hkl

Structure factors: contains datablock(s) II. DOI: 10.1107/S2056989022009100/yk2176IIsup5.hkl

CCDC references: 2206989, 2206988

Additional supporting information:  crystallographic information; 3D view; checkCIF report

## Figures and Tables

**Figure 9:**
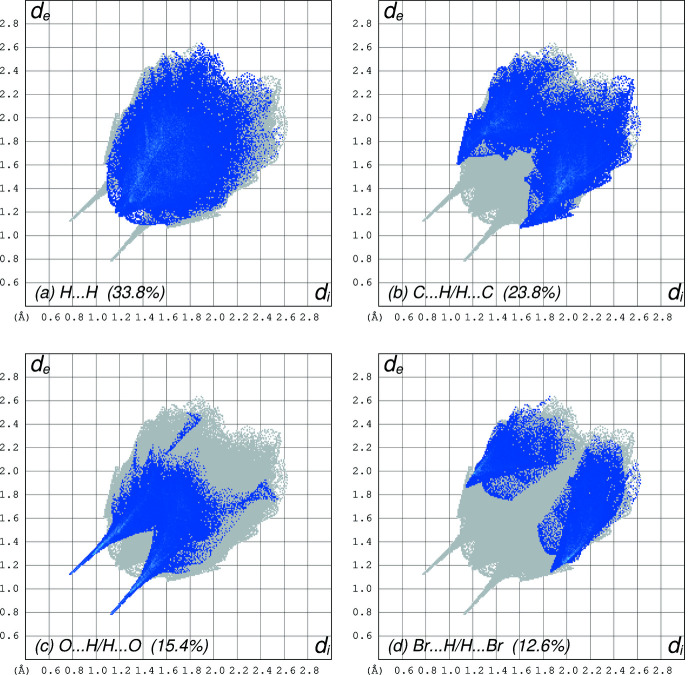
Fingerprint plots obtained from a Hirshfeld surface analysis for **II** using *CrystalExplorer*, separated into (*a*) H⋯H (33.8% coverage), (*b*) C⋯H/H⋯C (23.8%), (*c*) O⋯H/H⋯O (15.4%), (*d*) Br⋯H/H⋯Br (12.6%). All other contacts are negligible.

**Table 1:** Hydrogen-bond geometry (Å, °) for **I** [Chem scheme1]

*D*—H⋯*A*	*D*—H	H⋯*A*	*D*⋯*A*	*D*—H⋯*A*
O1—H1*O*⋯N1	0.90 (3)	1.73 (3)	2.546 (2)	148 (2)
N2—H2*N*⋯O1^i^	0.87 (2)	1.97 (2)	2.8225 (19)	168 (2)
C7—H7⋯O3^ii^	0.95	2.43	3.271 (2)	147

Symmetry codes: (i) 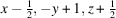 ; (ii) 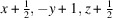 .

**Table 2:** Hydrogen-bond geometry (Å, °) for **II** [Chem scheme1]

*D*—H⋯*A*	*D*—H	H⋯*A*	*D*⋯*A*	*D*—H⋯*A*
O1*A*—H1*AO*⋯N1*A*	0.82 (2)	1.81 (2)	2.565 (2)	151 (2)
N2*A*—H2*AN*⋯O1*A* ^i^	0.87 (2)	2.04 (2)	2.902 (2)	171 (2)
C3*A*—H3*A*⋯O2*A* ^ii^	0.95	2.50	3.392 (2)	156
C6*A*—H6*A*⋯O3*A* ^i^	0.95	2.38	3.296 (2)	161
O1*B*—H1*BO*⋯N1*B*	0.80 (2)	1.84 (2)	2.558 (2)	148 (2)
N2*B*—H2*BN*⋯O1*B* ^iii^	0.88 (2)	2.04 (2)	2.915 (2)	171 (2)
C3*B*—H3*B*⋯O2*B* ^iv^	0.95	2.44	3.360 (2)	164
C6*B*—H6*B*⋯O3*B* ^iii^	0.95	2.39	3.297 (2)	159

Symmetry codes: (i) 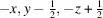 ; (ii) 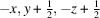 ; (iii) 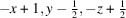 ; (iv) 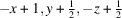 .

**Table 3:** Selected torsion angles (°) for **I**, **II*A*** and **II*B***

**I**			
C8—O2—C9—C10	−78.01 (18)	O2—C9—C10—C11	−59.9 (2)
**II**			
C8*A*—O2*A*—C9*A*—C10*A*	−98.12 (19)	O2*A*—C9*A*—C10*A*—C11*A*	−88.3 (2)
C8*B*—O2*B*—C9*B*—C10*B*	−98.0 (2)	O2*B*—C9*B*—C10*B*—C11*B*	−84.5 (2)

The above torsion angles qu­antify the most substantive differences between the conformations of **I**, **II*A*** and **II*B***.

**Table 4:** A sample of structures similar to **I** and **II** in the CSD *R* and *R*′ represent groups attached at the equivalent of C4 and *R*′′ represents the group attached at the equivalent of O3.

CSD refcode	*R*	*R*′	*R*′′	Reference
HODLOC	2-hy­droxy­phen­yl	H	meth­yl	Sun & Cheng (2008 ▸)
QOFLAZ	2-hy­droxy­phen­yl	H	eth­yl	Gao (2008 ▸)
KODVUV	4-hy­droxy­phen­yl	H	meth­yl	Cheng (2008*a* ▸)
XOGVEV	phen­yl	meth­yl	meth­yl	Cheng (2008*b* ▸)
XOGXEX	4-hy­droxy­phen­yl	H	eth­yl	Cheng (2008*c* ▸)
XOGXIB	3-meth­oxy-4-hy­droxy­phen­yl	H	meth­yl	Cheng (2008*d* ▸)
AZOTAL	3-hy­droxy­phen­yl	H	meth­yl	Li *et al.* (2011 ▸)
AWUJAE	3-hy­droxy­phen­yl	H	eth­yl	Hu *et al.* (2011 ▸)
WEFRUX	4-di­ethyl­amino-2-hy­droxy­phen­yl	H	meth­yl	Lv *et al.* (2017 ▸)

**Table 5:** Experimental details

	**I**	**II**
Crystal data		
Chemical formula	C_15_H_14_N_2_O_3_	C_15_H_13_BrN_2_O_3_
*M*_r_	270.28	349.18
Crystal system, space group	Monoclinic, *P* *n*	Monoclinic, *P*2_1_/*c*
Temperature (K)	90	90
*a*, *b*, *c* (Å)	4.5017 (12), 14.047 (4), 10.567 (3)	27.904 (2), 11.1207 (6), 9.0648 (7)
β (°)	96.300 (15)	94.485 (2)
*V* (Å^3^)	664.2 (3)	2804.3 (3)
*Z*	2	8
Radiation type	Cu *K*α	Mo *K*α
μ (mm^−1^)	0.79	2.94
Crystal size (mm)	0.41 × 0.23 × 0.02	0.24 × 0.22 × 0.05

Data collection		
Diffractometer	Bruker D8 Venture dual source	Bruker D8 Venture dual source
Absorption correction	Multi-scan (*SADABS*; Krause *et al.*, 2015 ▸)	Multi-scan (*SADABS*; Krause *et al.*, 2015 ▸)
*T*_min_, *T*_max_	0.589, 0.958	0.598, 0.862
No. of measured, independent and observed [*I* > 2σ(*I*)] reflections	7271, 2511, 2425	36145, 6401, 5004
*R*_int_	0.028	0.047
(sin θ/λ)_max_ (Å^−1^)	0.625	0.650

Refinement		
*R*[*F* ^2^ > 2σ(*F* ^2^)], *wR*(*F* ^2^), *S*	0.024, 0.063, 1.04	0.028, 0.064, 1.03
No. of reflections	2511	6401
No. of parameters	187	391
No. of restraints	2	0
H-atom treatment	H atoms treated by a mixture of independent and constrained refinement	H atoms treated by a mixture of independent and constrained refinement
Δρ_max_, Δρ_min_ (e Å^−3^)	0.13, −0.13	0.36, −0.39
Absolute structure	Flack *x* determined using 1054 quotients [(*I* ^+^)−(*I* ^−^)]/[(*I* ^+^)+(*I* ^−^)] (Parsons *et al.*, 2013 ▸)	–
Absolute structure parameter	0.04 (10)	–

Computer programs: *APEX3* (Bruker, 2016 ▸), *SHELXT* (Sheldrick, 2015*a* ▸), *SHELXL2019/2* (Sheldrick, 2015*b* ▸), *XP* in *SHELXTL* (Sheldrick, 2008 ▸), *CIFFIX* (Parkin, 2013 ▸) and *publCIF* (Westrip, 2010 ▸).

## supplementary crystallographic information

### Benzyl N'-[(E)-2-hydroxybenzylidene]hydrazinecarboxylate (I) Crystal data

**Table:**

C_15_H_14_N_2_O_3_	*F*(000) = 284
*M**_r_* = 270.28	*D*_x_ = 1.351 Mg m^−^^3^
Monoclinic, *P**n*	Cu *K*α radiation, λ = 1.54184 Å
*a* = 4.5017 (12) Å	Cell parameters from 6841 reflections
*b* = 14.047 (4) Å	θ = 3.1–74.4°
*c* = 10.567 (3) Å	µ = 0.79 mm^−^^1^
β = 96.300 (15)°	*T* = 90 K
*V* = 664.2 (3) Å^3^	Plate, colourless
*Z* = 2	0.41 × 0.23 × 0.02 mm

### Benzyl N'-[(E)-2-hydroxybenzylidene]hydrazinecarboxylate (I) Data collection

**Table:**

Bruker D8 Venture dual source diffractometer	2511 independent reflections
Radiation source: microsource	2425 reflections with *I* > 2σ(*I*)
Detector resolution: 7.41 pixels mm^-1^	*R*_int_ = 0.028
φ and ω scans	θ_max_ = 74.6°, θ_min_ = 3.2°
Absorption correction: multi-scan (*SADABS*; Krause *et al.*, 2015)	*h* = −5→5
*T*_min_ = 0.589, *T*_max_ = 0.958	*k* = −17→16
7271 measured reflections	*l* = −13→12

### Benzyl N'-[(E)-2-hydroxybenzylidene]hydrazinecarboxylate (I) Refinement

**Table:**

Refinement on *F*^2^	Secondary atom site location: difference Fourier map
Least-squares matrix: full	Hydrogen site location: mixed
*R*[*F*^2^ > 2σ(*F*^2^)] = 0.024	H atoms treated by a mixture of independent and constrained refinement
*wR*(*F*^2^) = 0.063	*w* = 1/[σ^2^(*F*_o_^2^) + (0.0283*P*)^2^ + 0.0531*P*] where *P* = (*F*_o_^2^ + 2*F*_c_^2^)/3
*S* = 1.04	(Δ/σ)_max_ < 0.001
2511 reflections	Δρ_max_ = 0.13 e Å^−^^3^
187 parameters	Δρ_min_ = −0.13 e Å^−^^3^
2 restraints	Absolute structure: Flack *x* determined using 1054 quotients [(*I*^+^)-(*I*^-^)]/[(*I*^+^)+(*I*^-^)] (Parsons *et al.*, 2013)
Primary atom site location: structure-invariant direct methods	Absolute structure parameter: 0.04 (10)

### Benzyl N'-[(E)-2-hydroxybenzylidene]hydrazinecarboxylate (I) Special details

**Table:**

Experimental. The crystal was mounted using polyisobutene oil on the tip of a fine glass fibre, which was fastened in a copper mounting pin with electrical solder. It was flash-cooled in liquid nitrogen and mounted into the cold gas stream of a liquid-nitrogen based cryostat using specially designed tongs (Parkin & Hope, 1998). Diffraction data were collected with the crystal at 90K, which is standard practice in this laboratory for the majority of flash-cooled crystals.
Geometry. All esds (except the esd in the dihedral angle between two l.s. planes) are estimated using the full covariance matrix. The cell esds are taken into account individually in the estimation of esds in distances, angles and torsion angles; correlations between esds in cell parameters are only used when they are defined by crystal symmetry. An approximate (isotropic) treatment of cell esds is used for estimating esds involving l.s. planes.
Refinement. Refinement progress was checked using *Platon* (Spek, 2020) and by an *R*-tensor (Parkin, 2000). The final model was further checked with the IUCr utility *checkCIF*.

### Benzyl N'-[(E)-2-hydroxybenzylidene]hydrazinecarboxylate (I) Fractional atomic coordinates and isotropic or equivalent isotropic displacement parameters (Å^2^)

**Table:**

	*x*	*y*	*z*	*U*_iso_*/*U*_eq_	
O1	0.7525 (3)	0.44387 (9)	0.34649 (11)	0.0268 (3)	
H1O	0.631 (6)	0.4786 (18)	0.391 (3)	0.040*	
O2	−0.0407 (3)	0.67535 (9)	0.56997 (11)	0.0243 (3)	
O3	0.2021 (3)	0.64243 (9)	0.39780 (11)	0.0248 (3)	
N1	0.4688 (3)	0.49495 (10)	0.53076 (13)	0.0202 (3)	
N2	0.2728 (3)	0.55477 (11)	0.58048 (13)	0.0214 (3)	
H2N	0.242 (5)	0.5508 (15)	0.660 (2)	0.026*	
C1	0.7887 (3)	0.36137 (13)	0.54751 (15)	0.0205 (3)	
C2	0.8612 (4)	0.37060 (12)	0.42217 (16)	0.0221 (4)	
C3	1.0493 (4)	0.30492 (14)	0.37311 (16)	0.0275 (4)	
H3	1.101603	0.312160	0.288959	0.033*	
C4	1.1602 (4)	0.22882 (14)	0.44739 (19)	0.0300 (4)	
H4	1.284895	0.183155	0.413092	0.036*	
C5	1.0907 (4)	0.21882 (14)	0.57132 (18)	0.0289 (4)	
H5	1.168389	0.166681	0.621816	0.035*	
C6	0.9082 (4)	0.28486 (13)	0.62116 (15)	0.0244 (4)	
H6	0.863234	0.278254	0.706480	0.029*	
C7	0.5866 (4)	0.42754 (12)	0.60123 (15)	0.0204 (3)	
H7	0.542993	0.420844	0.686734	0.025*	
C8	0.1509 (4)	0.62582 (12)	0.50506 (15)	0.0196 (3)	
C9	−0.1607 (4)	0.76046 (13)	0.50546 (17)	0.0251 (4)	
H9A	−0.343944	0.780464	0.542188	0.030*	
H9B	−0.215620	0.746317	0.414103	0.030*	
C10	0.0630 (4)	0.83981 (12)	0.51860 (17)	0.0231 (4)	
C11	0.1698 (4)	0.87256 (14)	0.63955 (19)	0.0311 (4)	
H11	0.100670	0.844554	0.712731	0.037*	
C12	0.3763 (5)	0.94578 (15)	0.6533 (2)	0.0401 (5)	
H12	0.448310	0.967698	0.736024	0.048*	
C13	0.4787 (5)	0.98720 (14)	0.5483 (3)	0.0418 (6)	
H13	0.619778	1.037661	0.558367	0.050*	
C14	0.3742 (5)	0.95472 (15)	0.4275 (2)	0.0390 (5)	
H14	0.444340	0.982844	0.354578	0.047*	
C15	0.1674 (4)	0.88122 (13)	0.41314 (18)	0.0285 (4)	
H15	0.096956	0.859121	0.330295	0.034*	

### Benzyl N'-[(E)-2-hydroxybenzylidene]hydrazinecarboxylate (I) Atomic displacement parameters (Å^2^)

**Table:**

	*U*^11^	*U*^22^	*U*^33^	*U*^12^	*U*^13^	*U*^23^
O1	0.0369 (8)	0.0313 (7)	0.0138 (5)	0.0058 (5)	0.0090 (5)	0.0020 (5)
O2	0.0269 (6)	0.0267 (6)	0.0202 (6)	0.0045 (5)	0.0066 (5)	0.0022 (5)
O3	0.0317 (6)	0.0292 (6)	0.0137 (5)	0.0005 (5)	0.0040 (4)	0.0007 (5)
N1	0.0235 (7)	0.0242 (7)	0.0132 (6)	0.0005 (5)	0.0041 (5)	−0.0019 (5)
N2	0.0259 (8)	0.0276 (8)	0.0117 (6)	0.0044 (6)	0.0064 (5)	−0.0001 (5)
C1	0.0217 (8)	0.0251 (8)	0.0148 (7)	−0.0008 (6)	0.0029 (6)	−0.0008 (6)
C2	0.0246 (8)	0.0262 (9)	0.0154 (7)	−0.0010 (7)	0.0025 (6)	−0.0011 (7)
C3	0.0322 (10)	0.0338 (10)	0.0172 (8)	0.0015 (7)	0.0062 (7)	−0.0041 (7)
C4	0.0304 (10)	0.0316 (10)	0.0285 (9)	0.0069 (8)	0.0053 (7)	−0.0079 (8)
C5	0.0313 (9)	0.0284 (9)	0.0265 (9)	0.0061 (7)	0.0009 (7)	0.0018 (7)
C6	0.0268 (8)	0.0290 (9)	0.0174 (8)	0.0020 (7)	0.0024 (7)	0.0011 (6)
C7	0.0233 (8)	0.0269 (8)	0.0114 (7)	−0.0004 (6)	0.0035 (6)	0.0001 (6)
C8	0.0212 (8)	0.0231 (8)	0.0144 (8)	−0.0021 (6)	0.0019 (6)	−0.0019 (6)
C9	0.0216 (8)	0.0278 (9)	0.0256 (8)	0.0037 (7)	0.0009 (7)	0.0018 (7)
C10	0.0194 (7)	0.0252 (9)	0.0243 (8)	0.0063 (6)	0.0009 (6)	−0.0018 (7)
C11	0.0254 (9)	0.0386 (11)	0.0290 (9)	0.0068 (8)	0.0010 (7)	−0.0067 (8)
C12	0.0287 (10)	0.0389 (11)	0.0503 (13)	0.0061 (8)	−0.0067 (9)	−0.0187 (9)
C13	0.0240 (9)	0.0252 (10)	0.0741 (16)	0.0034 (8)	−0.0039 (10)	−0.0034 (10)
C14	0.0279 (10)	0.0339 (11)	0.0552 (12)	0.0039 (8)	0.0042 (9)	0.0155 (10)
C15	0.0251 (9)	0.031 (1)	0.0290 (9)	0.0055 (7)	0.0012 (7)	0.0053 (7)

### Benzyl N'-[(E)-2-hydroxybenzylidene]hydrazinecarboxylate (I) Geometric parameters (Å, º)

**Table:**

O1—C2	1.361 (2)	C5—H5	0.9500
O1—H1O	0.90 (3)	C6—H6	0.9500
O2—C8	1.352 (2)	C7—H7	0.9500
O2—C9	1.451 (2)	C9—C10	1.498 (2)
O3—C8	1.204 (2)	C9—H9A	0.9900
N1—C7	1.283 (2)	C9—H9B	0.9900
N1—N2	1.365 (2)	C10—C15	1.384 (3)
N2—C8	1.355 (2)	C10—C11	1.393 (3)
N2—H2N	0.87 (2)	C11—C12	1.383 (3)
C1—C6	1.399 (2)	C11—H11	0.9500
C1—C2	1.405 (2)	C12—C13	1.376 (4)
C1—C7	1.459 (2)	C12—H12	0.9500
C2—C3	1.390 (3)	C13—C14	1.388 (4)
C3—C4	1.386 (3)	C13—H13	0.9500
C3—H3	0.9500	C14—C15	1.387 (3)
C4—C5	1.387 (3)	C14—H14	0.9500
C4—H4	0.9500	C15—H15	0.9500
C5—C6	1.381 (3)		
			
C2—O1—H1O	107.5 (17)	O3—C8—O2	125.21 (16)
C8—O2—C9	114.27 (13)	O3—C8—N2	126.12 (17)
C7—N1—N2	118.33 (14)	O2—C8—N2	108.67 (14)
C8—N2—N1	117.65 (14)	O2—C9—C10	110.94 (13)
C8—N2—H2N	121.3 (14)	O2—C9—H9A	109.5
N1—N2—H2N	120.8 (15)	C10—C9—H9A	109.5
C6—C1—C2	118.71 (16)	O2—C9—H9B	109.5
C6—C1—C7	119.42 (15)	C10—C9—H9B	109.5
C2—C1—C7	121.85 (15)	H9A—C9—H9B	108.0
O1—C2—C3	118.50 (16)	C15—C10—C11	119.09 (18)
O1—C2—C1	121.20 (16)	C15—C10—C9	121.48 (16)
C3—C2—C1	120.30 (16)	C11—C10—C9	119.43 (17)
C4—C3—C2	119.79 (17)	C12—C11—C10	120.1 (2)
C4—C3—H3	120.1	C12—C11—H11	119.9
C2—C3—H3	120.1	C10—C11—H11	119.9
C3—C4—C5	120.55 (17)	C13—C12—C11	120.7 (2)
C3—C4—H4	119.7	C13—C12—H12	119.7
C5—C4—H4	119.7	C11—C12—H12	119.7
C6—C5—C4	119.83 (17)	C12—C13—C14	119.5 (2)
C6—C5—H5	120.1	C12—C13—H13	120.2
C4—C5—H5	120.1	C14—C13—H13	120.2
C5—C6—C1	120.81 (16)	C15—C14—C13	120.0 (2)
C5—C6—H6	119.6	C15—C14—H14	120.0
C1—C6—H6	119.6	C13—C14—H14	120.0
N1—C7—C1	118.70 (14)	C10—C15—C14	120.53 (19)
N1—C7—H7	120.7	C10—C15—H15	119.7
C1—C7—H7	120.7	C14—C15—H15	119.7
			
C7—N1—N2—C8	179.79 (15)	C9—O2—C8—O3	−7.2 (2)
C6—C1—C2—O1	−179.68 (16)	C9—O2—C8—N2	173.15 (13)
C7—C1—C2—O1	2.1 (2)	N1—N2—C8—O3	−1.1 (3)
C6—C1—C2—C3	−0.3 (2)	N1—N2—C8—O2	178.58 (13)
C7—C1—C2—C3	−178.51 (16)	C8—O2—C9—C10	−78.01 (18)
O1—C2—C3—C4	−179.13 (17)	O2—C9—C10—C15	119.80 (18)
C1—C2—C3—C4	1.4 (3)	O2—C9—C10—C11	−59.9 (2)
C2—C3—C4—C5	−1.5 (3)	C15—C10—C11—C12	0.3 (3)
C3—C4—C5—C6	0.3 (3)	C9—C10—C11—C12	−179.98 (17)
C4—C5—C6—C1	0.9 (3)	C10—C11—C12—C13	0.1 (3)
C2—C1—C6—C5	−0.9 (3)	C11—C12—C13—C14	−0.3 (3)
C7—C1—C6—C5	177.40 (16)	C12—C13—C14—C15	0.2 (3)
N2—N1—C7—C1	178.01 (14)	C11—C10—C15—C14	−0.4 (3)
C6—C1—C7—N1	−177.06 (15)	C9—C10—C15—C14	179.89 (17)
C2—C1—C7—N1	1.2 (2)	C13—C14—C15—C10	0.2 (3)

### Benzyl N'-[(E)-2-hydroxybenzylidene]hydrazinecarboxylate (I) Hydrogen-bond geometry (Å, º)

**Table:**

*D*—H···*A*	*D*—H	H···*A*	*D*···*A*	*D*—H···*A*
O1—H1*O*···N1	0.90 (3)	1.73 (3)	2.546 (2)	148 (2)
N2—H2*N*···O1^i^	0.87 (2)	1.97 (2)	2.8225 (19)	168 (2)
C7—H7···O3^ii^	0.95	2.43	3.271 (2)	147

Symmetry codes: (i) *x*−1/2, −*y*+1, *z*+1/2; (ii) *x*+1/2, −*y*+1, *z*+1/2.

### Benzyl N'-[(E)-5-bromo-2-hydroxybenzylidene]hydrazinecarboxylate (II) Crystal data

**Table:**

C_15_H_13_BrN_2_O_3_	*F*(000) = 1408
*M**_r_* = 349.18	*D*_x_ = 1.654 Mg m^−^^3^
Monoclinic, *P*2_1_/*c*	Mo *K*α radiation, λ = 0.71073 Å
*a* = 27.904 (2) Å	Cell parameters from 9936 reflections
*b* = 11.1207 (6) Å	θ = 2.3–27.5°
*c* = 9.0648 (7) Å	µ = 2.94 mm^−^^1^
β = 94.485 (2)°	*T* = 90 K
*V* = 2804.3 (3) Å^3^	Plate, colourless
*Z* = 8	0.24 × 0.22 × 0.05 mm

### Benzyl N'-[(E)-5-bromo-2-hydroxybenzylidene]hydrazinecarboxylate (II) Data collection

**Table:**

Bruker D8 Venture dual source diffractometer	6401 independent reflections
Radiation source: microsource	5004 reflections with *I* > 2σ(*I*)
Detector resolution: 7.41 pixels mm^-1^	*R*_int_ = 0.047
φ and ω scans	θ_max_ = 27.5°, θ_min_ = 2.0°
Absorption correction: multi-scan (*SADABS*; Krause *et al*., 2015)	*h* = −36→36
*T*_min_ = 0.598, *T*_max_ = 0.862	*k* = −13→14
36145 measured reflections	*l* = −11→11

### Benzyl N'-[(E)-5-bromo-2-hydroxybenzylidene]hydrazinecarboxylate (II) Refinement

**Table:**

Refinement on *F*^2^	Primary atom site location: structure-invariant direct methods
Least-squares matrix: full	Secondary atom site location: difference Fourier map
*R*[*F*^2^ > 2σ(*F*^2^)] = 0.028	Hydrogen site location: mixed
*wR*(*F*^2^) = 0.064	H atoms treated by a mixture of independent and constrained refinement
*S* = 1.03	*w* = 1/[σ^2^(*F*_o_^2^) + (0.024*P*)^2^ + 0.4336*P*] where *P* = (*F*_o_^2^ + 2*F*_c_^2^)/3
6401 reflections	(Δ/σ)_max_ = 0.001
391 parameters	Δρ_max_ = 0.36 e Å^−^^3^
0 restraints	Δρ_min_ = −0.39 e Å^−^^3^

### Benzyl N'-[(E)-5-bromo-2-hydroxybenzylidene]hydrazinecarboxylate (II) Special details

**Table:**

Experimental. The crystal was mounted using polyisobutene oil on the tip of a fine glass fibre, which was fastened in a copper mounting pin with electrical solder. It was placed directly into the cold gas stream of a liquid-nitrogen based cryostat (Parkin & Hope, 1998). Diffraction data were collected with the crystal at 90K, which is standard practice in this laboratory for the majority of flash-cooled crystals.
Geometry. All esds (except the esd in the dihedral angle between two l.s. planes) are estimated using the full covariance matrix. The cell esds are taken into account individually in the estimation of esds in distances, angles and torsion angles; correlations between esds in cell parameters are only used when they are defined by crystal symmetry. An approximate (isotropic) treatment of cell esds is used for estimating esds involving l.s. planes.
Refinement. Refinement progress was checked using *Platon* (Spek, 2020) and by an *R*-tensor (Parkin, 2000). The final model was further checked with the IUCr utility *checkCIF*.

### Benzyl N'-[(E)-5-bromo-2-hydroxybenzylidene]hydrazinecarboxylate (II) Fractional atomic coordinates and isotropic or equivalent isotropic displacement parameters (Å^2^)

**Table:**

	*x*	*y*	*z*	*U*_iso_*/*U*_eq_	
Br1A	−0.15724 (2)	0.00040 (2)	−0.27291 (2)	0.01837 (6)	
O1A	−0.03689 (5)	0.32791 (12)	0.13515 (17)	0.0155 (3)	
H1AO	−0.0212 (8)	0.285 (2)	0.195 (3)	0.023*	
O2A	0.07623 (4)	0.09899 (12)	0.57652 (16)	0.0147 (3)	
O3A	0.05320 (5)	0.27304 (12)	0.45841 (16)	0.0164 (3)	
N1A	0.00001 (5)	0.13879 (14)	0.25999 (19)	0.0131 (4)	
N2A	0.03016 (6)	0.08825 (15)	0.3687 (2)	0.0144 (4)	
H2AN	0.0326 (8)	0.011 (2)	0.378 (3)	0.022*	
C1A	−0.05776 (6)	0.12521 (17)	0.0571 (2)	0.0123 (4)	
C2A	−0.06384 (6)	0.25104 (17)	0.0464 (2)	0.0128 (4)	
C3A	−0.09807 (7)	0.29968 (17)	−0.0559 (2)	0.0151 (4)	
H3A	−0.102322	0.384378	−0.061072	0.018*	
C4A	−0.12600 (7)	0.22594 (18)	−0.1503 (2)	0.0161 (4)	
H4A	−0.149393	0.259472	−0.220254	0.019*	
C5A	−0.11949 (6)	0.10144 (17)	−0.1417 (2)	0.0135 (4)	
C6A	−0.08603 (7)	0.05145 (17)	−0.0399 (2)	0.0133 (4)	
H6A	−0.082137	−0.033375	−0.035397	0.016*	
C7A	−0.02400 (7)	0.07044 (17)	0.1679 (2)	0.0135 (4)	
H7A	−0.019897	−0.014331	0.171790	0.016*	
C8A	0.05341 (7)	0.16443 (17)	0.4676 (2)	0.0134 (4)	
C9A	0.11033 (7)	0.16320 (18)	0.6787 (2)	0.0159 (4)	
H9A1	0.108034	0.132746	0.780527	0.019*	
H9A2	0.102462	0.250018	0.677625	0.019*	
C10A	0.16046 (7)	0.14533 (18)	0.6338 (2)	0.0152 (4)	
C11A	0.18757 (7)	0.04758 (18)	0.6867 (3)	0.0199 (5)	
H11A	0.175224	−0.004677	0.757468	0.024*	
C12A	0.23246 (7)	0.0256 (2)	0.6372 (3)	0.0244 (5)	
H12A	0.250802	−0.041479	0.673897	0.029*	
C13A	0.25050 (7)	0.1014 (2)	0.5344 (3)	0.0236 (5)	
H13A	0.280911	0.084987	0.498459	0.028*	
C14A	0.22449 (7)	0.2012 (2)	0.4832 (3)	0.0234 (5)	
H14A	0.237391	0.254413	0.414556	0.028*	
C15A	0.17947 (7)	0.22296 (18)	0.5330 (2)	0.0184 (5)	
H15A	0.161541	0.291233	0.498007	0.022*	
Br1B	0.33187 (2)	0.24274 (2)	−0.24668 (2)	0.01864 (6)	
O1B	0.46666 (5)	0.56485 (12)	0.12357 (18)	0.0175 (3)	
H1BO	0.4824 (8)	0.526 (2)	0.183 (3)	0.026*	
O2B	0.58093 (5)	0.33767 (12)	0.56327 (17)	0.0200 (3)	
O3B	0.55787 (5)	0.51149 (12)	0.44468 (17)	0.0213 (3)	
N1B	0.50017 (6)	0.37664 (15)	0.2571 (2)	0.0163 (4)	
N2B	0.53006 (6)	0.32678 (15)	0.3664 (2)	0.0177 (4)	
H2BN	0.5316 (8)	0.248 (2)	0.381 (3)	0.027*	
C1B	0.44047 (7)	0.36259 (17)	0.0590 (2)	0.0144 (4)	
C2B	0.43755 (7)	0.48817 (17)	0.0413 (2)	0.0155 (4)	
C3B	0.40395 (7)	0.53794 (18)	−0.0621 (2)	0.0187 (5)	
H3B	0.402207	0.622759	−0.073766	0.022*	
C4B	0.37308 (7)	0.46506 (18)	−0.1480 (2)	0.0186 (5)	
H4B	0.349893	0.499454	−0.217909	0.022*	
C5B	0.37609 (7)	0.34063 (18)	−0.1317 (2)	0.0157 (4)	
C6B	0.40953 (7)	0.28942 (18)	−0.0301 (2)	0.0153 (4)	
H6B	0.411493	0.204431	−0.020934	0.018*	
C7B	0.47346 (7)	0.30758 (17)	0.1725 (2)	0.0159 (5)	
H7B	0.475081	0.222686	0.183473	0.019*	
C8B	0.55672 (7)	0.40391 (18)	0.4573 (2)	0.0164 (4)	
C9B	0.61708 (7)	0.40024 (19)	0.6607 (3)	0.0199 (5)	
H9B1	0.616291	0.369612	0.762983	0.024*	
H9B2	0.609811	0.487367	0.661257	0.024*	
C10B	0.66606 (7)	0.38057 (18)	0.6078 (2)	0.0177 (5)	
C11B	0.69188 (7)	0.27764 (19)	0.6499 (3)	0.0243 (5)	
H11B	0.678700	0.220613	0.713444	0.029*	
C12B	0.73692 (8)	0.2581 (2)	0.5992 (3)	0.0314 (6)	
H12B	0.754349	0.187171	0.627110	0.038*	
C13B	0.75634 (8)	0.3413 (2)	0.5085 (3)	0.0339 (6)	
H13B	0.787039	0.327312	0.473303	0.041*	
C14B	0.73134 (8)	0.4452 (2)	0.4685 (3)	0.0302 (6)	
H14B	0.745077	0.503149	0.407352	0.036*	
C15B	0.68621 (7)	0.4646 (2)	0.5178 (2)	0.0216 (5)	
H15B	0.668973	0.535692	0.489816	0.026*	

### Benzyl N'-[(E)-5-bromo-2-hydroxybenzylidene]hydrazinecarboxylate (II) Atomic displacement parameters (Å^2^)

**Table:**

	*U*^11^	*U*^22^	*U*^33^	*U*^12^	*U*^13^	*U*^23^
Br1A	0.02056 (10)	0.01578 (11)	0.01793 (13)	−0.00204 (8)	−0.00389 (8)	−0.00260 (9)
O1A	0.0167 (7)	0.0096 (7)	0.0194 (9)	−0.0001 (5)	−0.0033 (6)	0.0007 (6)
O2A	0.0156 (7)	0.0133 (7)	0.0146 (9)	−0.0002 (5)	−0.0031 (6)	−0.0003 (6)
O3A	0.0195 (7)	0.0107 (7)	0.0193 (9)	−0.0015 (5)	0.0025 (6)	−0.0002 (6)
N1A	0.0121 (8)	0.0127 (8)	0.0143 (11)	0.0018 (6)	0.0009 (7)	0.0020 (7)
N2A	0.0173 (8)	0.0093 (8)	0.0159 (11)	0.0011 (7)	−0.0025 (7)	0.0005 (7)
C1A	0.0124 (9)	0.0113 (9)	0.0134 (12)	0.0006 (7)	0.0033 (8)	0.0007 (8)
C2A	0.0120 (9)	0.0116 (9)	0.0153 (12)	−0.0028 (8)	0.0051 (8)	−0.0014 (8)
C3A	0.0155 (10)	0.0102 (9)	0.0196 (13)	0.0018 (8)	0.0019 (9)	0.0018 (8)
C4A	0.015 (1)	0.0161 (10)	0.0172 (13)	0.0025 (8)	0.0018 (8)	0.0035 (9)
C5A	0.0113 (9)	0.0159 (10)	0.0132 (12)	−0.0019 (8)	−0.0002 (8)	−0.0021 (8)
C6A	0.0158 (10)	0.0101 (9)	0.0145 (12)	−0.0009 (8)	0.0049 (8)	0.0007 (8)
C7A	0.0145 (9)	0.0100 (9)	0.0164 (13)	0.0005 (7)	0.0042 (8)	0.0005 (8)
C8A	0.0109 (9)	0.0134 (10)	0.0164 (12)	−0.0005 (7)	0.0045 (8)	−0.0003 (8)
C9A	0.0176 (10)	0.0171 (10)	0.0125 (12)	−0.0022 (8)	−0.0018 (9)	−0.0042 (8)
C10A	0.0155 (10)	0.0172 (10)	0.0124 (12)	−0.0013 (8)	−0.0029 (8)	−0.0060 (8)
C11A	0.0235 (11)	0.0167 (10)	0.0185 (13)	−0.0030 (9)	−0.0049 (9)	0.0007 (9)
C12A	0.0185 (11)	0.0253 (12)	0.0278 (15)	0.0061 (9)	−0.0083 (9)	−0.0055 (10)
C13A	0.0137 (10)	0.0311 (13)	0.0257 (15)	−0.0020 (9)	0.0000 (9)	−0.0118 (10)
C14A	0.0211 (11)	0.0254 (12)	0.0237 (14)	−0.0073 (9)	0.0026 (10)	−0.0037 (10)
C15A	0.0214 (10)	0.0154 (10)	0.0176 (13)	−0.0007 (8)	−0.0029 (9)	−0.0026 (9)
Br1B	0.0178 (1)	0.01828 (11)	0.01947 (13)	−0.00271 (8)	−0.00094 (8)	−0.00299 (9)
O1B	0.0174 (7)	0.0116 (7)	0.0227 (10)	−0.0013 (6)	−0.0037 (6)	−0.0002 (6)
O2B	0.0174 (7)	0.0154 (7)	0.0259 (10)	−0.0012 (6)	−0.0070 (6)	−0.0003 (6)
O3B	0.0230 (7)	0.0118 (7)	0.0284 (10)	−0.0024 (6)	−0.0017 (7)	−0.0006 (6)
N1B	0.0153 (9)	0.0139 (9)	0.0193 (12)	0.0015 (6)	−0.0009 (8)	0.0029 (7)
N2B	0.0183 (9)	0.0115 (8)	0.0221 (12)	0.0006 (7)	−0.0058 (8)	0.0015 (8)
C1B	0.0126 (9)	0.0146 (10)	0.0162 (12)	0.0002 (8)	0.0031 (8)	0.0001 (8)
C2B	0.0137 (9)	0.0133 (10)	0.0200 (13)	−0.0021 (8)	0.0034 (8)	−0.0029 (9)
C3B	0.0197 (10)	0.0124 (10)	0.0243 (14)	0.0013 (8)	0.0028 (9)	0.0013 (9)
C4B	0.0189 (10)	0.0177 (10)	0.0189 (13)	0.0015 (8)	−0.0004 (9)	0.0017 (9)
C5B	0.0125 (9)	0.0172 (10)	0.0176 (13)	−0.0023 (8)	0.0037 (8)	−0.0034 (9)
C6B	0.0167 (10)	0.0111 (10)	0.0186 (13)	−0.0006 (8)	0.0039 (9)	−0.0004 (8)
C7B	0.0152 (10)	0.0102 (9)	0.0222 (13)	0.0003 (8)	0.0009 (9)	0.0003 (8)
C8B	0.0132 (10)	0.0165 (10)	0.0197 (13)	0.0002 (8)	0.0024 (8)	0.0011 (9)
C9B	0.018 (1)	0.0211 (11)	0.0195 (13)	−0.0016 (8)	−0.0056 (9)	−0.0039 (9)
C10B	0.0174 (10)	0.0190 (11)	0.0160 (13)	−0.0011 (8)	−0.0033 (9)	−0.0051 (9)
C11B	0.0229 (11)	0.0182 (11)	0.0307 (15)	−0.0014 (9)	−0.0041 (10)	−0.002 (1)
C12B	0.0240 (12)	0.0267 (13)	0.0419 (17)	0.0059 (10)	−0.0069 (11)	−0.0121 (12)
C13B	0.0198 (12)	0.0505 (16)	0.0320 (17)	−0.0009 (11)	0.0052 (11)	−0.0142 (13)
C14B	0.0285 (12)	0.0410 (15)	0.0213 (15)	−0.0098 (11)	0.0042 (10)	−0.0048 (11)
C15B	0.0255 (11)	0.0225 (11)	0.0160 (13)	−0.0015 (9)	−0.0032 (9)	−0.0018 (9)

### Benzyl N'-[(E)-5-bromo-2-hydroxybenzylidene]hydrazinecarboxylate (II) Geometric parameters (Å, º)

**Table:**

Br1A—C5A	1.8949 (19)	Br1B—C5B	1.896 (2)
O1A—C2A	1.360 (2)	O1B—C2B	1.360 (2)
O1A—H1AO	0.82 (2)	O1B—H1BO	0.80 (2)
O2A—C8A	1.346 (2)	O2B—C8B	1.349 (2)
O2A—C9A	1.461 (2)	O2B—C9B	1.464 (2)
O3A—C8A	1.211 (2)	O3B—C8B	1.203 (2)
N1A—C7A	1.279 (2)	N1B—C7B	1.282 (2)
N1A—N2A	1.365 (2)	N1B—N2B	1.362 (2)
N2A—C8A	1.361 (3)	N2B—C8B	1.369 (3)
N2A—H2AN	0.87 (2)	N2B—H2BN	0.88 (2)
C1A—C6A	1.399 (3)	C1B—C6B	1.397 (3)
C1A—C2A	1.412 (3)	C1B—C2B	1.407 (3)
C1A—C7A	1.456 (3)	C1B—C7B	1.460 (3)
C2A—C3A	1.388 (3)	C2B—C3B	1.388 (3)
C3A—C4A	1.381 (3)	C3B—C4B	1.378 (3)
C3A—H3A	0.9500	C3B—H3B	0.9500
C4A—C5A	1.398 (3)	C4B—C5B	1.394 (3)
C4A—H4A	0.9500	C4B—H4B	0.9500
C5A—C6A	1.378 (3)	C5B—C6B	1.381 (3)
C6A—H6A	0.9500	C6B—H6B	0.9500
C7A—H7A	0.9500	C7B—H7B	0.9500
C9A—C10A	1.500 (3)	C9B—C10B	1.499 (3)
C9A—H9A1	0.9900	C9B—H9B1	0.9900
C9A—H9A2	0.9900	C9B—H9B2	0.9900
C10A—C11A	1.388 (3)	C10B—C15B	1.388 (3)
C10A—C15A	1.392 (3)	C10B—C11B	1.390 (3)
C11A—C12A	1.385 (3)	C11B—C12B	1.389 (3)
C11A—H11A	0.9500	C11B—H11B	0.9500
C12A—C13A	1.380 (3)	C12B—C13B	1.377 (4)
C12A—H12A	0.9500	C12B—H12B	0.9500
C13A—C14A	1.386 (3)	C13B—C14B	1.384 (4)
C13A—H13A	0.9500	C13B—H13B	0.9500
C14A—C15A	1.389 (3)	C14B—C15B	1.385 (3)
C14A—H14A	0.9500	C14B—H14B	0.9500
C15A—H15A	0.9500	C15B—H15B	0.9500
			
C2A—O1A—H1AO	105.5 (16)	C2B—O1B—H1BO	107.9 (17)
C8A—O2A—C9A	116.62 (15)	C8B—O2B—C9B	116.93 (16)
C7A—N1A—N2A	119.20 (16)	C7B—N1B—N2B	119.03 (17)
C8A—N2A—N1A	117.02 (16)	N1B—N2B—C8B	117.14 (17)
C8A—N2A—H2AN	121.5 (15)	N1B—N2B—H2BN	122.0 (15)
N1A—N2A—H2AN	121.3 (15)	C8B—N2B—H2BN	120.8 (15)
C6A—C1A—C2A	118.64 (18)	C6B—C1B—C2B	118.97 (18)
C6A—C1A—C7A	119.38 (17)	C6B—C1B—C7B	119.38 (18)
C2A—C1A—C7A	121.97 (18)	C2B—C1B—C7B	121.59 (18)
O1A—C2A—C3A	118.04 (17)	O1B—C2B—C3B	117.61 (18)
O1A—C2A—C1A	121.65 (18)	O1B—C2B—C1B	122.20 (18)
C3A—C2A—C1A	120.30 (18)	C3B—C2B—C1B	120.18 (18)
C4A—C3A—C2A	120.53 (18)	C4B—C3B—C2B	120.41 (19)
C4A—C3A—H3A	119.7	C4B—C3B—H3B	119.8
C2A—C3A—H3A	119.7	C2B—C3B—H3B	119.8
C3A—C4A—C5A	119.28 (18)	C3B—C4B—C5B	119.61 (19)
C3A—C4A—H4A	120.4	C3B—C4B—H4B	120.2
C5A—C4A—H4A	120.4	C5B—C4B—H4B	120.2
C6A—C5A—C4A	121.02 (18)	C6B—C5B—C4B	120.81 (18)
C6A—C5A—Br1A	119.71 (14)	C6B—C5B—Br1B	120.43 (15)
C4A—C5A—Br1A	119.27 (15)	C4B—C5B—Br1B	118.72 (15)
C5A—C6A—C1A	120.22 (18)	C5B—C6B—C1B	120.00 (18)
C5A—C6A—H6A	119.9	C5B—C6B—H6B	120.0
C1A—C6A—H6A	119.9	C1B—C6B—H6B	120.0
N1A—C7A—C1A	118.65 (17)	N1B—C7B—C1B	118.39 (18)
N1A—C7A—H7A	120.7	N1B—C7B—H7B	120.8
C1A—C7A—H7A	120.7	C1B—C7B—H7B	120.8
O3A—C8A—O2A	126.12 (19)	O3B—C8B—O2B	126.55 (19)
O3A—C8A—N2A	125.18 (19)	O3B—C8B—N2B	125.7 (2)
O2A—C8A—N2A	108.70 (16)	O2B—C8B—N2B	107.76 (17)
O2A—C9A—C10A	109.76 (16)	O2B—C9B—C10B	109.90 (17)
O2A—C9A—H9A1	109.7	O2B—C9B—H9B1	109.7
C10A—C9A—H9A1	109.7	C10B—C9B—H9B1	109.7
O2A—C9A—H9A2	109.7	O2B—C9B—H9B2	109.7
C10A—C9A—H9A2	109.7	C10B—C9B—H9B2	109.7
H9A1—C9A—H9A2	108.2	H9B1—C9B—H9B2	108.2
C11A—C10A—C15A	119.15 (19)	C15B—C10B—C11B	119.4 (2)
C11A—C10A—C9A	120.30 (19)	C15B—C10B—C9B	120.74 (19)
C15A—C10A—C9A	120.48 (18)	C11B—C10B—C9B	119.9 (2)
C12A—C11A—C10A	120.6 (2)	C12B—C11B—C10B	120.1 (2)
C12A—C11A—H11A	119.7	C12B—C11B—H11B	119.9
C10A—C11A—H11A	119.7	C10B—C11B—H11B	119.9
C13A—C12A—C11A	119.9 (2)	C13B—C12B—C11B	120.0 (2)
C13A—C12A—H12A	120.1	C13B—C12B—H12B	120.0
C11A—C12A—H12A	120.1	C11B—C12B—H12B	120.0
C12A—C13A—C14A	120.4 (2)	C12B—C13B—C14B	120.3 (2)
C12A—C13A—H13A	119.8	C12B—C13B—H13B	119.9
C14A—C13A—H13A	119.8	C14B—C13B—H13B	119.9
C13A—C14A—C15A	119.6 (2)	C13B—C14B—C15B	119.8 (2)
C13A—C14A—H14A	120.2	C13B—C14B—H14B	120.1
C15A—C14A—H14A	120.2	C15B—C14B—H14B	120.1
C14A—C15A—C10A	120.4 (2)	C14B—C15B—C10B	120.4 (2)
C14A—C15A—H15A	119.8	C14B—C15B—H15B	119.8
C10A—C15A—H15A	119.8	C10B—C15B—H15B	119.8
			
C7A—N1A—N2A—C8A	−177.37 (18)	C7B—N1B—N2B—C8B	−177.79 (18)
C6A—C1A—C2A—O1A	−178.68 (17)	C6B—C1B—C2B—O1B	−179.66 (18)
C7A—C1A—C2A—O1A	3.0 (3)	C7B—C1B—C2B—O1B	3.1 (3)
C6A—C1A—C2A—C3A	1.6 (3)	C6B—C1B—C2B—C3B	0.8 (3)
C7A—C1A—C2A—C3A	−176.79 (18)	C7B—C1B—C2B—C3B	−176.38 (19)
O1A—C2A—C3A—C4A	179.10 (18)	O1B—C2B—C3B—C4B	−179.38 (19)
C1A—C2A—C3A—C4A	−1.2 (3)	C1B—C2B—C3B—C4B	0.2 (3)
C2A—C3A—C4A—C5A	0.0 (3)	C2B—C3B—C4B—C5B	−0.7 (3)
C3A—C4A—C5A—C6A	0.6 (3)	C3B—C4B—C5B—C6B	0.2 (3)
C3A—C4A—C5A—Br1A	−179.20 (15)	C3B—C4B—C5B—Br1B	178.02 (16)
C4A—C5A—C6A—C1A	−0.2 (3)	C4B—C5B—C6B—C1B	0.8 (3)
Br1A—C5A—C6A—C1A	179.65 (14)	Br1B—C5B—C6B—C1B	−176.99 (15)
C2A—C1A—C6A—C5A	−0.9 (3)	C2B—C1B—C6B—C5B	−1.3 (3)
C7A—C1A—C6A—C5A	177.49 (18)	C7B—C1B—C6B—C5B	175.98 (19)
N2A—N1A—C7A—C1A	177.06 (16)	N2B—N1B—C7B—C1B	178.03 (17)
C6A—C1A—C7A—N1A	−176.88 (18)	C6B—C1B—C7B—N1B	−177.23 (19)
C2A—C1A—C7A—N1A	1.5 (3)	C2B—C1B—C7B—N1B	0.0 (3)
C9A—O2A—C8A—O3A	−11.2 (3)	C9B—O2B—C8B—O3B	−8.9 (3)
C9A—O2A—C8A—N2A	169.09 (15)	C9B—O2B—C8B—N2B	171.53 (16)
N1A—N2A—C8A—O3A	−7.7 (3)	N1B—N2B—C8B—O3B	−4.0 (3)
N1A—N2A—C8A—O2A	171.98 (15)	N1B—N2B—C8B—O2B	175.60 (16)
C8A—O2A—C9A—C10A	−98.12 (19)	C8B—O2B—C9B—C10B	−98.0 (2)
O2A—C9A—C10A—C11A	−88.3 (2)	O2B—C9B—C10B—C15B	95.9 (2)
O2A—C9A—C10A—C15A	88.5 (2)	O2B—C9B—C10B—C11B	−84.5 (2)
C15A—C10A—C11A—C12A	−1.6 (3)	C15B—C10B—C11B—C12B	−1.5 (3)
C9A—C10A—C11A—C12A	175.19 (19)	C9B—C10B—C11B—C12B	178.9 (2)
C10A—C11A—C12A—C13A	0.0 (3)	C10B—C11B—C12B—C13B	0.8 (4)
C11A—C12A—C13A—C14A	1.7 (3)	C11B—C12B—C13B—C14B	0.6 (4)
C12A—C13A—C14A—C15A	−1.8 (3)	C12B—C13B—C14B—C15B	−1.1 (4)
C13A—C14A—C15A—C10A	0.1 (3)	C13B—C14B—C15B—C10B	0.4 (3)
C11A—C10A—C15A—C14A	1.6 (3)	C11B—C10B—C15B—C14B	1.0 (3)
C9A—C10A—C15A—C14A	−175.24 (19)	C9B—C10B—C15B—C14B	−179.5 (2)

### Benzyl N'-[(E)-5-bromo-2-hydroxybenzylidene]hydrazinecarboxylate (II) Hydrogen-bond geometry (Å, º)

**Table:**

*D*—H···*A*	*D*—H	H···*A*	*D*···*A*	*D*—H···*A*
O1*A*—H1*AO*···N1*A*	0.82 (2)	1.81 (2)	2.565 (2)	151 (2)
N2*A*—H2*AN*···O1*A*^i^	0.87 (2)	2.04 (2)	2.902 (2)	171 (2)
C3*A*—H3*A*···O2*A*^ii^	0.95	2.50	3.392 (2)	156
C6*A*—H6*A*···O3*A*^i^	0.95	2.38	3.296 (2)	161
O1*B*—H1*BO*···N1*B*	0.80 (2)	1.84 (2)	2.558 (2)	148 (2)
N2*B*—H2*BN*···O1*B*^iii^	0.88 (2)	2.04 (2)	2.915 (2)	171 (2)
C3*B*—H3*B*···O2*B*^iv^	0.95	2.44	3.360 (2)	164
C6*B*—H6*B*···O3*B*^iii^	0.95	2.39	3.297 (2)	159

Symmetry codes: (i) −*x*, *y*−1/2, −*z*+1/2; (ii) −*x*, *y*+1/2, −*z*+1/2; (iii) −*x*+1, *y*−1/2, −*z*+1/2; (iv) −*x*+1, *y*+1/2, −*z*+1/2.
